# Improved Gene Transfer with Functionalized Hollow Mesoporous Silica Nanoparticles of Reduced Cytotoxicity

**DOI:** 10.3390/ma10070731

**Published:** 2017-06-30

**Authors:** Zhengwen Zhan, Xiaoxu Zhang, Jiayuan Huang, Ying Huang, Zhengwei Huang, Xin Pan, Guilan Quan, Hu Liu, Lili Wang, Chuanbin Wu

**Affiliations:** 1 School of Pharmaceutical Sciences, Sun Yat-Sen University, Guangzhou 510006, China; zhanzhw@mail2.sysu.edu.cn (Z.Z.); zhangxiaoxu@yeah.net (X.Z.); jiayuan.huang@monash.edu (J.H.); huangy2007@163.com (Y.H.); hzhengw3@mail2.sysu.edu.cn (Z.H.); mercurypan@foxmail.com (X.P.); chuanbin_wu@126.com (C.W.); 2 Zhongshan WanYuan New Drug R&D Co., Ltd., Zhongshan 528451, China; 3 School of Pharmacy, Memorial University of Newfoundland, Newfoundland and Labrador, St. John’s, NL A1B 3V6, Canada; hliu@mun.ca (H.L.); lwang@mun.ca (L.W.)

**Keywords:** hollow mesoporous silica nanoparticles, PEI, gene transfer, cytotoxicity

## Abstract

Gene therapy is a promising strategy for treatment of genetically caused diseases. Successful gene delivery requires an efficient carrier to transfer the desired gene into host cells. Recently, mesoporous silica nanoparticles (MSNs) functionalized with 25 kD polyethyleneimine (PEI) were extensively used as gene delivery carriers. However, 25 kD PEI could significantly reduce the safety of the modified MSNs although it is efficient for intracellular delivery of nucleic acids. In addition, limited drug loading remains a challenge for conventional MSNs drug carriers. Hollow mesoporous silica nanoparticles (HMSNs) with high pore volume, tunable pore size, and excellent biocompatibility are attractive alternatives. To make them more efficient, a less toxic 1.8 kD PEI polymer was used to functionalize the HMSNs which have large pore size (~10 nm) and form PEI-HMSNs. Scanning and transmission electron microscopic images showed that HMSNs were spherical in shape and approximately 270 nm in diameter with uniform hollow nanostructures. The maximum loading capacity of green fluorescent protein labeled DNA (GFP-DNA) in PEI-HMSNs was found to be 37.98 mg/g. The loading capacity of PEI-HMSNs was nearly three-fold higher than those of PEI modified solid nanoparticles, indicating that both hollow and large pores contributed to the increase in DNA adsorption. The transfection of GFP-DNA plasmid loaded in PEI-HMSNs was increased two-fold in comparison to that of 25 kD PEI. MTT assays in Lovo cells showed that the cell viability was more than 85% when the concentration of PEI-HMSNs was 120 µg/mL, whereas the cell viability was less than 20% when the 25 kD PEI was used at the same concentration. These results indicated that PEI-HMSNs could be used as a delivery system for nucleic acids due to good biocompatibility, high gene loading capacity, and enhanced gene transfer efficiency.

## 1. Introduction

Gene therapy is a promising strategy to cure a wide range of inherited diseases, such as cancer, AIDS, and cardiovascular disease. However, transferring a desired gene into host cell nucleus to treat genetic disorders is a complex process with several restrictions. Successful gene delivery requires an efficient system to deliver genes into specific cells, since naked nucleic acids alone can be rapidly degraded by endogenous nucleases and hard to penetrate through cell membranes [1]. Therefore, an effective gene delivery vector should be able to load a sufficient amount of genes, provide a protective environment, and effectively penetrate into cells. In addition, the preparation for an ideal gene carrier should be easy and the carrier system must be non-toxic.

Currently, there are two main systems for gene delivery: viral and non-viral vector systems. Generally, the viral vector system was the most common and simple one for gene delivery where non-pathogenic virus—including retro-, adeno-, and adeno-associated viral vectors—are used [2]. However, several limitations—including the risk of provoking immune response, high cost, preparation difficulty, and limited availability of genetic materials that can be inserted into human cells—have restricted the use of these vectors in gene therapy [3].

Thus, recent research activities have focused on the development of cheaper and safer non-viral vector systems, including liposomes, cation polymers, and inorganic porous nanoparticles. Though liposomes have been widely studied for improved transfection, their physicochemical instability and undesirable gene leakage have impeded their further applications. Cationic polymers such as polyethyleneimines (PEIs) have attracted much attention as gene delivery carriers, since they contain protonation of amino and could enhance gene delivery via “proton sponge effect” on cells [4,5]. However, their transfection efficiency is positively correlated with the molecular weight as well as the cytotoxicity [6,7]. In contrast, inorganic porous nanoparticles possess many advantages such as large surface area, negligible cytotoxicity, and cost-effective preparation. Owing to their better gene delivery efficiency, inorganic porous nanoparticles combined with cationic substances have been extensively studied recently [8].

Among these systems, mesoporous silica nanoparticles (MSNs) [9] are particularly attractive due to the easiness of surface functionalization, which includes surface texture exploitation and pore size adjustment for surface area and pore volume control, particularly exploitation of the surface charge [10,11]. MSNs functionalized with PEIs are likely to adsorb DNA molecules more effectively due to their positive charge and subsequently protect the nucleic acids from degradation from endogenous nucleases [12,13]. However, the use of high molecular weight PEIs (such as 10 kD and 25 kD) would significantly reduce the safety of modified MSNs although it is efficient for intracellular delivery [14,15]. This is likely due to the positive charge of MSN-PEI complexes, which results in a strong electrostatic interaction with the negatively charged cell membrane leading to high cellular uptake and thus elevated cell death.

Therefore, balancing transfection efficiency and cytotoxicity is an essential aspect for gene delivery [16]. Hollow mesoporous silica nanoparticles (HMSNs) turn out as a promising solution to above mentioned challenges because of their hollow inner structure and higher outside surface area [15,17,18]. In this study, HMSNs was integrated with low molecular weight PEI (1.8 kD PEI) to construct a novel gene delivery system. The inner pores of HMSNs were also functionalized with PEI to enhance the DNA adsorption. The obtained system was expected to enhance gene transfer efficiency and to minimize cytotoxicity.

## 2. Results and Discussion

### 2.1. Synthesis and Characterization of HMSNs

**Figure 1:**
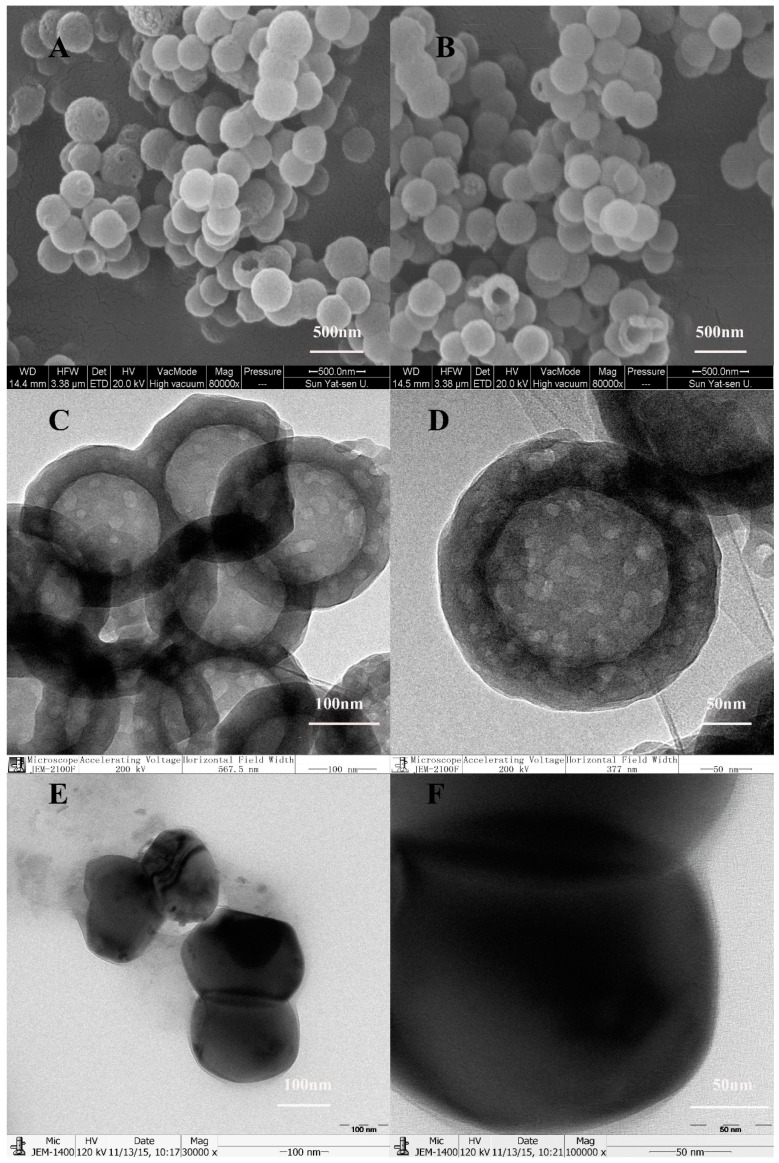
The morphology of nanoparticles. (**A**,**B**): SEM images of HMSNs; (**C**,**D**): TEM images of HMSNs; (**E**,**F**): TEM images of as-synthesized HMSNs without removing the template.

HMSNs were synthesized according to a procedure previously reported [19]. The scanning electron microscope (SEM) images (Figure 1A,B) and transmission electron microscopy (TEM) images (Figure 1C,D) showed that HMSNs were nearly spherical in shape (~270 nm in diameter) with uniformly distributed hollow nanostructures. The bright color showing the cores of the nanoparticles (Figure 1C,D) is an indication of hollow cores.

**Figure 2:**
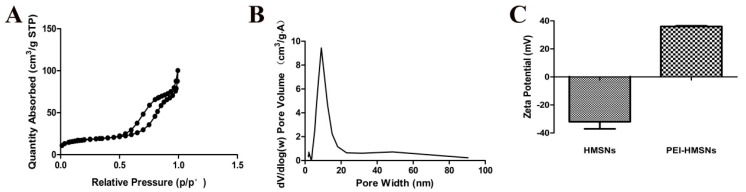
Characterization of porous structure and surface charge. (**A**): Nitrogen adsorption/desorption isotherms of HMSNs; (**B**): Pore size distribution in HMSNs; (**C**): Zeta potentials of HMSNs and PEI-HMSNs.

Since the mesoporous shell structure was made of tetraethyl orthosilicate (TEOS) and octadecyltrimethoxysilane (C_18_TMS), vertical nanochannels connecting to the internal core would be generated when C_18_TMS is completely removed. The gene was likely to be loaded and released through these nanochannels. The TEM images (Figure 1E,F) showed that as-synthesized HMSNs without etching and calcination process appeared as a black solid, spherical in shape without any hollow cavity and nanostructures. In contrast, both the inner core and the outer shell of the calcined HMSNs were translucent, indicating the generated hollow cavity and mesopores (Figure 1C,D). In order to obtain more precise information about the structure of HMSNs, nitrogen adsorption–desorption measurements were performed. According to the International Union of Pure and Applied Chemistry classification, the results of N_2_ adsorption–desorption isotherms (Figure 2A) suggested that HMSNs possessed the typical Langmuir IV hysteresis loops, conforming the existence of mesopores with a narrow distribution of pore size in a range of 5.0–20.0 nm and a sharp peak at around 10.0 nm (Figure 2B). The Brunauer–Emmett–Teller (BET) surface area, pore volume, and average pore size were calculated as 84.27 m^2^/g, 19.36 cm^3^/g and 10 nm, respectively. The BET surface area was lower than other MSNs, this might be ascribed to the inner space and the relatively large pore size which suggested a potential for high gene loading.

Cationic polyelectrolyte PEIs contain potentially protonated amino groups and are found effective in gene delivery due to their “proton sponge” effect for its strong escape ability from acidic endosomes in cells [20]. To promote gene binding affinity and transfection efficiency, positively charged 1.8 kD PEI was used to modify the negatively charged HMSNs. Modifications on the inner surface are critical to increase the loading of gene material and to shield DNA from degradation. The zeta potentials of PEI-HMSNs and HMSNs were found to be 36.00 ± 0.47 mV and −32.00 ± 5.01 mV, respectively, indicating that 1.8 kD PEI was successfully attached onto the HMSNs (Figure 2C).

### 2.2. DNA Adsorption Ability of PEI-HMSNs

**Figure 3:**
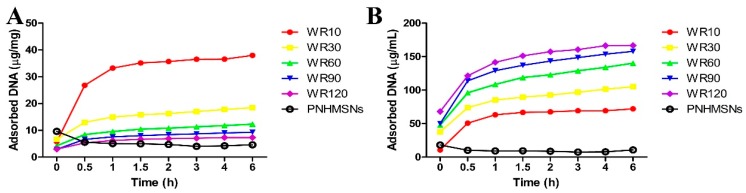
The DNA adsorption by PEI-HMSNs. (**A**): DNA adsorption isotherms by PEI-HMSNs with various PEI-HMSNs/DNA weight ratios and PNHMSNs/DNA at WR60; (**B**): Total DNA (µg/mL) in the sediment after incubating DNA with PEI-HMSNs and PHMSNs/DNA at WR60 was calculated by subtracting DNA in the supernatant determined at different time intervals from initial total DNA added.

Green fluorescent protein labeled DNA (GFP-DNA) was used to evaluate the gene adsorption ability of PEI-HMSNs by incubating different amounts of PEI-HMSNs with a fixed amount of GFP-DNA (DNA-PEI-HMSNs). Solberg, S.M., et al. [21] reported that MSNs with pores greater than 5.4 nm were found to be favorable for DNA (2000 bp) absorption into the inter pores. In this study, the pore size (10 nm) was large enough to allow GFP-DNA (760 bp) to diffuse into the hollow cavity. The solid nanoparticles (NHMSNs) with identical particle size without etching and calcination were used as a control. The amount of PEI bound to these solid nanoparticles (without pores/hollow space) was also controlled to be same as the hollow MSNs. Loading capability was calculated as a ratio of the DNA weight loaded into PEI-HMSNs to the weight of DNA added. The results are shown in Figure 3 when the weight ratio (WR) of PEI-HMSNs to GFP-DNA was fixed at 10, 30, 60, 90, and 120, respectively. The positively charged PEI-HMSNs were likely bonded with the negatively charged GFP-DNA through electrostatic interaction. After incubation for 2 h, the GFP-DNA encapsulated in the sediment reached the maximum value. It was shown that the amount of GFP-DNA loaded in PEI-HMSNs per gram increased as the WR of PEI-HMSNs to GFP-DNA decreased (Figure 3A). The least amount of DNA was loaded in PEI-HMSNs when the WR of PEI-HMSNs/GFP-DNA was at 120, while the WR of 10 resulted in the highest loading capacity (37.98 µg/mg). Similarly, the total DNA adsorbed on PEI-HMSNs increased as the WR decreased (Figure 3B). In contrast, the DNA adsorption capacity of PNHMSNs was relatively low. Only 4.61 µg/mg of DNA was adsorbed when the WR of PNHMSNs/GFP-DNA was 60, which was much lower than that of PEI-HMSNs at the same WR (12.32 µg/mg). Additionally, Sean M. Solberg et al. also used amino propyl-modified MSNs without hollow structure with increased particle size (2 µm in diameter) and higher surface area [21]. However, the maximum loading capacity of PEI-HMSNs in our study was found to be two-fold greater than their system. Therefore, the results clearly demonstrated that both hollow space and large pores contributed to significantly increase the DNA loading, and the pores could enable DNA to be loaded into the hollow cavity.

### 2.3. Cytotoxicity of PEI-HMSNs

**Figure 4:**
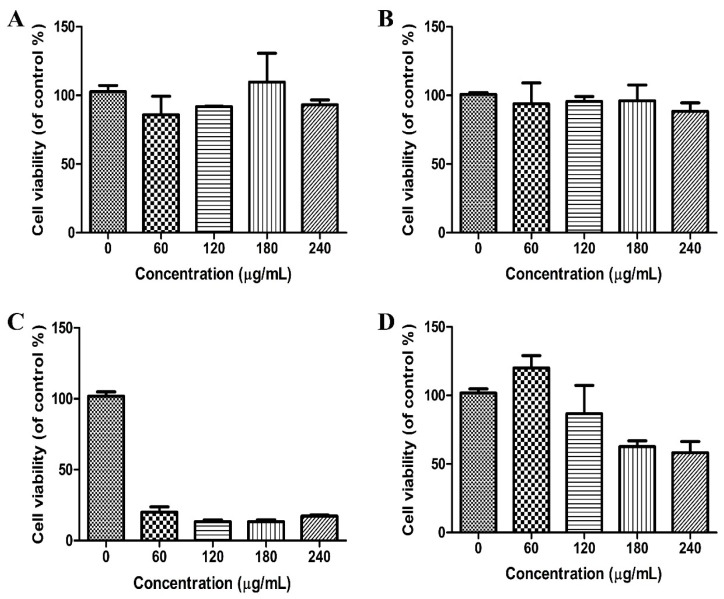
Cell viabilities of Lovo cells upon incubation with (**A**): 1.8 kD PEI; (**B**): HMSNs; (**C**): 25 kD PEI; and (**D**): PEI-HMSNs for 24 h, respectively. The number of independent determinations was at least three.

The biosafety of the carriers must be taken into consideration before application. While PEIs are effective for complexing and delivering genes, polymer-based delivery systems often lead to cytotoxicity due to the damage to the cell membrane and subcellular components like lysosomes and mitochondria [22]. Meanwhile, Zink et al. reported that the cytotoxicity of PEI can be modified by adjusting its chain length [16]. PEIs of longer lengths seem to possess increased ability in damaging the cell membrane and cellular components, so that they show stronger ability of cell uptake with higher cytotoxicity. This probably explains the cytotoxicity observed with the 25 kD PEI. To evaluate the safety of PEI-HMSNs, a commonly used cytotoxicity determination method, 3-(4,5-dimethylthiazol-2-yl)-2,5-diphenyltetrazoliumbromide (MTT) assay, was used. Lovo cells were incubated with HMSNs, 1.8 kD PEI, and PEI-HMSNs at concentrations of 0, 60, 120, 180, and 240 µg/mL, respectively. In comparison, 25 kD PEI which is known to be cytotoxic, was used. The results of MTT assay are shown in Figure 4. Lovo cells showed good viability upon incubation with 1.8 kD PEI and HMSNs, up to 240 µg/mL for 24 h (Figure 4A,B). In contrast, significant cytotoxicity was observed with 25 kD PEI over the same concentration range and the cell viability dropped to less than 20% when the concentration of 25 kD PEI was as low as 60 µg/mL (Figure 4C). The cytotoxicity of 1.8 kD PEI was obviously lower than 25 kD PEI.

The cytotoxicity of PEI-HMSNs was found to be concentration-dependent (Figure 4D). However, the cell viability of PEI-HMSNs was still found to be higher than 85% when its concentration was 120 µg/mL as shown in Figure 4D.

### 2.4. Gene Transfer Efficiency and Cellular Uptake

**Figure 5:**
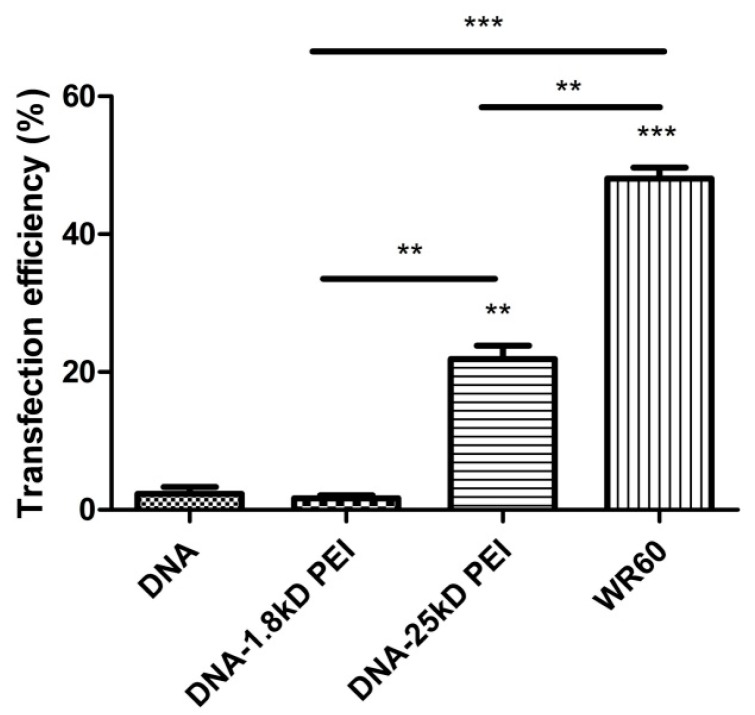
In vitro transfection efficiency of Lovo cells. The WR of PEI-HMSNs to DNA was fixed at 60 and the number of independent determinations was at least three (**, *** *p* < 0.05).

Flow cytometer study was employed to evaluate the cellular transfection efficiency of DNA-PEI-HMSNs. In consideration of the loading capacity and cytotoxicity of PEI-HMSNs, PEI-HMSNs/DNA at a ratio of 60 (or 120 µg PEI-HMSN_S_/2 µg DNA) was chosen in the gene transfer experiment. The transfection results are shown in Figure 5, and the gated transfection of the naked GFP-DNA, DNA-1.8 kD PEI, DNA-25 kD PEI, and DNA-PEI-HMSNs were 2.30, 1.67, 21.89, and 48.06, respectively. The transfection of DNA-PEI-HMSNs is two-fold greater than that of DNA-25 kD PEI while DNA-1.8 kD PEI and naked GFP-DNA showed negligible transfection efficiency. These results indicated that PEI-HMSNs nanoparticles could promote the accumulation of DNA in cells by the endocytosis to facilitate the transfection process, while both naked DNA and DNA-1.8 kD PEI complex could not get through the cell membrane of Lovo cells.

**Figure 6:**
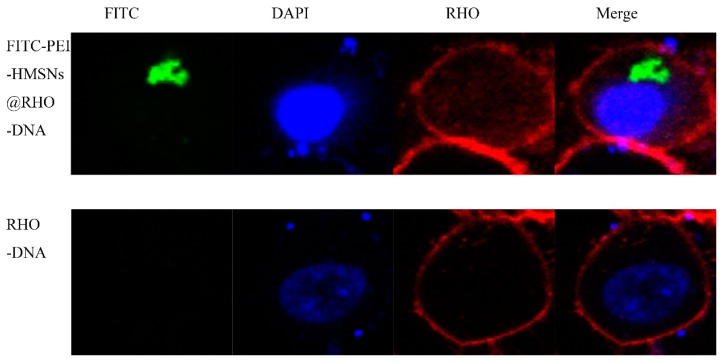
Confocal microscopy images of Lovo cells incubated with RHO-DNA and FITC-PEI-HMSNs for 4 h at 37 °C. Cell nuclei were stained blue with DAPI, RHO-DNA were stained red with rhodamine phalloidin, and FITC was shown as green fluorescence.

Cellular uptake of nanoparticles was studied by tracking the distribution of fluorescent DNA-PEI-HMSNs. Fluorescein isothiocyanate (FITC) is an electrophile and it normally reacts with nucleophiles such as hydroxyl and amino groups [23]. However, FITC was unable to bind with HMSNs directly likely due to the calcination process which might have destroyed the FITC on the surface. Therefore, a different method was explored. FITC was first bonded with a fraction of PEI’s amino groups and then this PEI-FITC fragment was combined with HMSNs through electrostatic attraction. Rhodamine labeled DNA (RHO-DNA) was loaded into FITC-PEI-HMSNs to produce a complex, FITC-PEI-HMSNs@RHO-DNA, for cellular uptake study. The Lovo cells were treated with the FITC-PEI-HMSNs@RHO-DNA complex followed with an additional 4 h culture. Results were observed using a laser scanning confocal microscope (Figure 6). It showed that Lovo cells treated with naked RHO-DNA presented a very weak RHO signal and only the blue color (nucleus) displayed after staining with 4′,6-diamidino-2-phenylindole (DAPI), indicating RHO-DNA was unable to be transfected alone into the cells. In contrast, various green fluorescent spots were observed when the Lovo cells were incubated with FITC-PEI-HMSNs@RHO-DNA complex, indicating successful uptake of FITC-PEI-HMSNs into cells, likely through endocytosis.

## 3. Materials and Methods

### 3.1. Materials

Tetraethyl orthosilicate (TEOS), ethanol, anhydrous sodium carbonate (Na_2_CO_3_), ammonia solution (25–28%, *v*/*v*), and C_18_TMS were purchased from Aladdin Co., Ltd. (Shanghai, China). PEIs with molecular weights of 1800 and 25,000 (i.e., 1.8 kD PEI and 25 kD PEI) were obtained from Alfa Aesar (Heysham, UK). GFP base pair sequence of the forward primer (5′ to 3′) was AGC TGC TAT GTT GTG TGG; the sequence of the reverse primer (3′ to 5′) was GTG GTC TCT CTT TTC GTT GG. MTT, DAPI and FITC were purchased from Sigma-Aldrich (St. Louis, MO, USA). Dulbecco’s Modified Eagle’s Medium (DMEM), trypsin-EDTA, fetal bovine serum (FBS), and penicillin streptomycin were acquired from GIBCO (Gaithersburg, MD, USA). All reagents used were of analytical grade unless otherwise specified.

Human colonic carcinoma cell line Lovo was obtained from Shanghai Institute of Cell Biology of Chinese Academy of Sciences (Shanghai, China). The cells were cultured in DMEM supplemented with 10% FBS (*v*/*v* %) and 100 U/mL of penicillin-streptomycin in a humidified incubator containing 5% CO_2_ at 37 °C. The media were changed every two days prior to the experiment.

### 3.2. Methods

#### 3.2.1. Synthesis of Large Pore-Sized HMSNs

The HMSNs were synthesized according to the following steps: preparation of monodisperse silica nanoparticles (NPs), coating mesoporous silica layer on the surface of NPs and subsequent alkaline etching in Na_2_CO_3_ solution for different time intervals. Briefly, ethanol (71.4 mL), deionized (DI) water (10 mL) and ammonia solution (3.14 mL) were mixed with magnetic stirring at 30 °C. TEOS (6 mL) was rapidly added into the mixture, followed by magnetic stirring for 1 h. TEOS (5 mL) premixed with C_18_TMS (3 mL) was then rapidly poured into the above solution and the mixture was stirred for another 1 h, the particles were collected by centrifugation (6000 rpm) for 5 min. The obtained product was dispersed in 0.6 M Na_2_CO_3_ solution (50 mL) which was kept at 80 °C with magnetic stirring for 10 h to obtain HMSNs of 10 nm pore size. The etched HMSNs were washed thoroughly with DI water to completely remove Na_2_CO_3_ in the solution. Finally, the preparation was dried under vacuum for 6 h and calcined at 550 °C to completely remove C_18_TMS.

#### 3.2.2. Characterization of HMSNs

The morphology of HMSNs was characterized using SEM (JSM-6330 F, JEOL, Tokyo, Japan). The samples were sputter-coated with gold prior to imaging. TEM images were acquired on a JEM-2100F electron microscope operating at an accelerating voltage of 200 kV and 120 kV. Zeta-potentials were measured using a Mavern Nanosizer. Brunauer–Emmett–Teller surface area, pore volume, and diameter distribution (N_2_ adsorption–desorption technique) of HMSNs were measured at −196 °C using a surface area and pore size analyzer (ASAP 2020C, Micromeritics, Norcross, GA, USA).

#### 3.2.3. Synthesis of GFP Labelled DNA, i.e., GFP-DNA

A solution of DI H_2_O (100 mL), Lysogeny Broth base (2.0 g), and ampicillin (1 mL of a 100 mg/L solution) was prepared in a 150 mL flask. It was then autoclaved and cooled to room temperature. Subsequently, 100 µL of ampicillin-resistant *E. coli* strain with a DNA plasmid containing the gene for GFP was added. The mixture was then incubated at 37 °C for 48 h. It was then refrigerated. The *E. coli* DNA was isolated and purified using a Wizard Plus Minipreps DNA Purification System (Promega, Shanghai, China).

#### 3.2.4. Preparation of PEI-HMSNs

Briefly, 5 mg of HMSNs were dispersed in a solution containing 2.5 mg of 1.8 kD PEI and 1 mL of absolute ethanol under stirring for 30 min, and then washed with ethanol and DI water, respectively.

#### 3.2.5. Preparation of FITC-PEI-HMSNs

FITC (8.4 mg) and 1.8 kD PEI (2.5 mg) were dispersed in 1.5 mL of absolute ethanol. The mixture was stirred for 4 h, followed by adding 5 mg of HMSNs and stirring for another 30 min. The obtained product was washed with ethanol and DI water, respectively.

#### 3.2.6. Preparation of DNA-PEI-HMSNs

Five different amounts of PEI-HMSNs (200, 600, 1200, 1800, and 2400 µg) and DNA (20 µg) were added to sterilized DI water to obtain five different PEI-HMSNs to DNA ratio samples (10, 30, 60, 90, and 120). The respective mixtures were incubated for 2 h at room temperature to induce the formation of DNA-PEI-HMSNs complex.

#### 3.2.7. DNA Loading Efficiency Test

Various amounts of PEI-HMSNs were mixed with DNA sample in 1 mL of DI water to achieve PEI-HMSNs to DNA weight ratio of 10, 30, 60, 90, and 120, respectively. PNHMSNs were mixed with DNA sample in 1 mL of DI water to achieve PNHMSNs to DNA weight ratio of 60. The respective mixtures were stirred for 30 min to allow the loading of DNA into the nanoparticles. The supernatant was collected by centrifugation at different time intervals. The amount of unloaded DNA in the supernatant was determined using a nanodrop 2000 UV–Vis spectrophotometer. The amount of DNA loaded in PEI-HMSNs and PNHMSNs was calculated by subtracting DNA in the supernatant from the initial DNA added. Three independent experiments were carried out for statistical analysis.

#### 3.2.8. Cytotoxicity Study

The cytotoxicity of 1.8 kD PEI, 25 kD PEI, HMSNs, and PEI-HMSNs was evaluated in Lovo cells using MTT assay. Cells were seeded in 96-well plates at a density of 2 × 10^4^ cells per well. After incubation in 5% CO_2_ at 37 °C for 24 h, the medium was replaced by 200 μL of fresh medium containing different samples. Cells treated with pure medium were used as the blank control. After incubation with the samples for 24 h, the medium was replaced with 180 μL of fresh medium and 20 μL of MTT (5 mg/mL), and incubated for additional 4 h at 37 °C. Then, 150 μL of DMSO was added in each well to dissolve the purple formazan crystals and the absorbance was measured at 490 nm using an ELX 800 micro-plate reader. The cytotoxicity was calculated as the percentage of cell viability as compared with the blank control, and results were expressed as mean ± standard deviation (SD) of three independent wells. Cell viability was calculated as the following formula: Relative cell viability = (absorbance of treated cells–bsorbance of blank)/(absorbance of control cells–absorbance of blank) × 100%.

#### 3.2.9. In Vitro Transfection Experiments

Cells were seeded in 12-well culture plates at a density of 2 × 10^5^ per well. After incubation in a 5% CO_2_ atmosphere at 37 °C for 24 h, the cells were 80% confluent. Then DNA-PEI-HMSNs prepared using 2 µg of DNA and 120 µg of PEI-HMSNs was added. After incubation at 37 °C for 4 h, the medium was removed and replaced by fresh complete culture medium, and the cells were allowed to culture for another two days. Finally, the cells were harvested and analyzed for GFP fluorescence expression by a flow cytometer (Beckman Coulter, Fullerton, CA, USA). In addition, DNA, DNA-1.8 kD PEI, and DNA-25 kD PEI were also examined.

#### 3.2.10. Confocal Microscopy Study

Lovo cells were seeded in the observation dishes and cultured at 37 °C for 24 h, then treated with fluorescence labelled samples, FITC-HMSNs and a rhodamine-labeled DNA (RHO-DNA), for additional 4 h. The medium was then removed and the cells were washed using cold PBS (pH 7.4) and fixed with 4% paraformaldehyde at room temperature for 15 min. Subsequently, the cell nuclei were labelled with DAPI for 15 min. Images were acquired using the laser scanning confocal microscope (LSCM, Zessi LSM710, Heidenheim, Germany).

## 4. Conclusions

PEI-HMSNs synthesized appears to be promising as a gene delivery platform, because of their larger surface area, higher pore volume, favorable pore size, excellent biocompatibility, and optimal stability. PEI-HMSNs was shown with morphological characteristics and loading capability more desirable for gene delivery. Ultimately, PEI-HMSNs encapsulated DNA was shown to be successful in the gene transfection experiment, which was confirmed by the confocal microscopy. In conclusion, we have successfully fabricated HMSNs coupled with 1.8 kD PEI as a carrier for encapsulation of GFP-DNA, which resulted in the transfer of GFP-DNA into the cell. The PEI-HMSNs exhibited significantly lower cytotoxicity in comparison to 25 kD PEI. These results suggest the potential of PEI-HMSNs in gene therapy and genetic engineering.
